# Diagnostic and therapeutic challenges of a heparin-induced thrombocytopenia in HIV immunodeficiency: in regards to a pediatric case (a case report)

**DOI:** 10.11604/pamj.2025.51.43.46466

**Published:** 2025-06-13

**Authors:** Mouna Zouine, Yousra El Boussadani, Abdallah Oulmaati

**Affiliations:** 1 Pediatrics and Neonatology Department, Mohammed VI University Hospital Center of Tangier, Tangier, Morocco,; 2 Faculty of Medicine and Pharmacy, Abdelmalek Essaâdi University, Tangier, Morocco

**Keywords:** Thrombocytopenia, cerebral venous thrombosis, anticoagulant treatment, case report

## Abstract

We are dealing with a female infant case who had a cerebral venous thrombosis, revealing a secondary immunodeficiency, owing to a human immunodeficiency virus infection. After anticoagulant treatment with low-molecular-weight heparin (LMWH), it came to light that she had a severe thrombocytopenia, suggesting a thrombocytopenia induced by an auto-immune origin of heparin. Within a developing country in which means of confirmation are not always available, it was difficult to identify whether severe thrombocytopenia was owing to HIV infection, sepsis, or heparin. The therapeutic decision was therefore difficult, because stopping anticoagulation can lead to a cerebral thrombosis extension.

## Introduction

The annual incidence of venous thrombosis of the newborn baby, particularly the cerebral one, is probably underrated, due to the discretion or the non-specificity of some symptoms, or when these are masked within a more general clinical picture [1]. Treatment is based on symptomatic, etiological, and especially antithrombotic one [2]. Nevertheless, in rare cases, the heparin anticoagulation may lead to a severe form of thrombocytopenia called heparin-induced thrombocytopenia (HIT). It is a rare iatrogenic disease known by its potential seriousness, mainly related to thrombosis, and by difficulties while performing diagnosis and care [3]. We report in this observation, the rough situation of a newborn baby affected with human immunodeficiency virus (HIV) with multiple cerebral venous thrombosis associated with hemorrhagic infarction, treated by low molecular weight heparin with the emergence of severe thrombocytopenia in the days following the initiation of anticoagulation and with whom the decision-making whether to stop or continue anticoagulant treatment was a therapeutic challenge.

## Patient and observation

**Patient information:** a three-month-old female baby, the youngest of her three siblings, with no particular pathological history. She was admitted for a seizure in a fever context associated with a progressive increase in the cranial circumference, noticed by her mother a week ago.

**Clinical findings:** the clinical examination at admission found a patient in stage II coma, pale, febrile, tachycardic, and polypneic. The cranial circumference was +2 SD, weight and height were normal as far as her age is concerned. Hepatomegaly was noted on the abdominal examination, with a condensation syndrome on the pulmonary examination.

**Figure 1:**
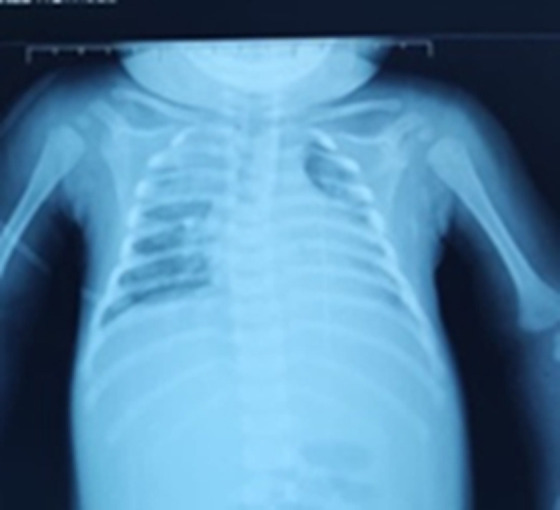
front chest X-ray in a supine position, demonstrating a systematized alveolar location of the right upper lobe associated with a scattered bilateral interstitial syndrome

**Figure 2:**
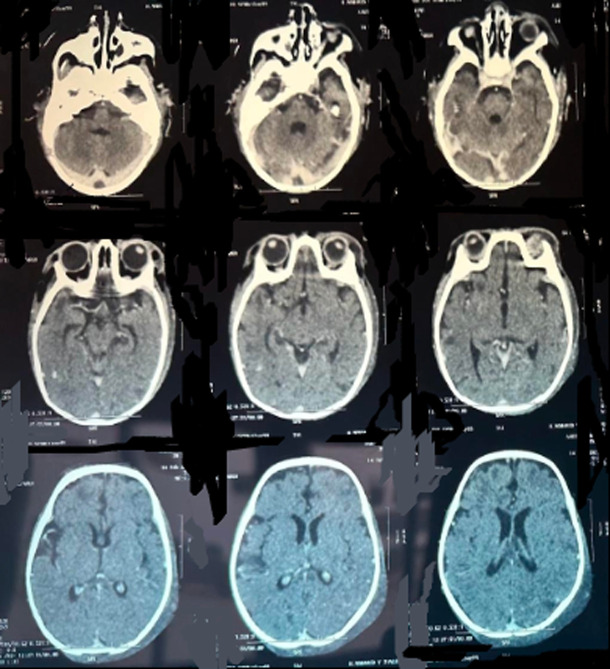
presence at the level of the bilateral frontal subarachnoid spaces and in the right temporo-occipital of well-limited tubular hyperdensities realizing the “cord sign”: harmonious aspect with a thrombus of the cortical veins, associated with bilateral hypodense foci presenting a hemorrhagic component: secondary infarction to thrombosis

**Diagnostic assessment:** the cerebral computed tomography (CT) scan (Figure 1) showed the presence at the level of tubular hyperdensity subarachnoid well-limited spaces, realizing the “cord sign” harmonious with a thrombus of the cortical veins, associated with bilateral hypodense foci with a hemorrhagic component. The set of these lesions was associated with edematous infiltration of soft tissues of the left parietal scalp related to a hematoma in the process of liquefaction. The chest X-ray (Figure 2) demonstrated a bilateral alveolo-interstitial syndrome. The complete blood count showed a microcytic hypochromic anemia at 4.9g/l, a leucopenia at 3,900/ul; a neutropenia at 1000, a lymphopenia at 1000, and thrombocytopenia at 107.000/ul. The CRP at 116 mg/l, a TP at 23%, TCK: 43 sec, ALT 779 IU/L, AST 500 IU/L, albuminemia: 27 g/L, urea 7.52 mmol/L, and creatinine: 31.82 ùmol/L. The hemoculture remained sterile, whereas the cytobacteriological study of sputum isolated a *Klebsiella pneumoniae*. Facing the seriousness of the clinical picture associated with the pancytopenia, the persistent lymphopenia, and the hepatocellular insufficiency, a rapid HIV test was positive. The viral load was 2,760,000 copies/ml, confirming the infection by the human immunodeficiency virus.

The low-molecular-weight heparin at a dose of 100 IU/kg/12h was associated with an anticonvulsant treatment with a red blood cells and fresh frozen plasma transfusions were initiated. An antibiotic therapy based on imipenem, amikacin, cotrimoxazole, and ganciclovir was also prescribed for the patient while waiting for the start of the antiretroviral treatment after the pre-therapeutic assessment.

**Diagnosis:** six days after starting the low-molecular-weight heparins (LMWHs), the evolution was obvious, with neurological deterioration and the appearance of severe thrombocytopenia at 27,000/uL. Given the suspicion of heparin-induced thrombocytopenia, an anti-PF4 antibody test has been requested.

**Therapeutic interventions:** in the face of the severe thrombocytopenia, the anticoagulation was stopped. The search for anti-F4P antibodies and the control cerebral imaging could not be carried out, giving the fact that the immediate access to these examinations remains difficult in our context.

**Follow-up and outcome of interventions:** twenty-four hours later, the newborn baby died, with a picture of severe sepsis and deterioration of the neurological state.

**Patient perspective:** in this case, it has been impossible to gather the patient's perspective, as the patient is an infant. Furthermore, the perspectives of the parents could not be specified due to the particular context of the illness. Indeed, the infant's HIV infection has revealed HIV carriage in both parents, which has complicated the possibility of obtaining a detailed view of their feelings regarding the situation.

**Informed consent:** consent for the publication of the case report and related data was obtained from both parents.

## Discussion

The cerebral venous thrombosis (CVT) is rare in children, but possibly has serious consequences, owing to the intracranial hypertension and parenchymal lesions. The thrombus may involve several contiguous venous segments, and its location varies according to the age of the child and the underlying pathology [4]. HIV infection is one of these pathologies that tends to promote the occurrence of thrombotic accidents. In this case, they are essentially due to the hypercoagulability state caused by HIV itself, and which is responsible for an immunodeficiency, hemostasis disorders, and a prolonged inflammatory state. Opportunistic infections, such as toxoplasmosis and cytomegalovirus (CMV), as well as the iatrogenic effects of antiretrovirals, also play a role in the occurrence of these thrombotic accidents [5].

Studies have suggested that protein S deficiency associated with HIV infection could also be involved [6]. In this newborn baby´s case, the TORSCH serologies, protein S dosage, and cerebral MRI could not be performed due to a lack of resources. However, this did not prevent us from starting the anticoagulation by LMWH, despite the haemorrhagic component, in order to avoid the thromboses extension and their complications. According to various studies [7,8], cerebral hemorrhagic sufficiency, which complicates venous hypertension upstream of the thrombus, does not constitute a contraindication to anticoagulant treatment, particularly the LMWH, which is firstly used. Nevertheless, this anticoagulant treatment is devoid of risk. Heparin-induced thrombocytopenia (HIT), which is of immune origin and potentially is fatal, is one of the side effects. It must be sought in the presence of any clinical suspicion by the 4T score and confirmed by the presence of anti-PF4/heparin antibodies [3].

As far as this observation is concerned, and according to the 4T score, the probability of a HIT was intermediate, and there were several factors that could explain the occurrence of such severe thrombocytopenia. In particular, in the state of sepsis and the immunosuppression field, a heparin-induced thrombocytopenia could not be discarded, because the coexistence of all these pathologies does not eliminate the diagnosis [3]. On the contrary, these abnormalities aggravate even more the HIT. The means to confirm the HIT is the search in the plasma for heparin-dependent IgG antibodies activating platelets and causing aggregation. Therefore, it is necessary to systematically conduct a search for anti-F4P antibodies and a functional test [3,9]. The inaccessibility to these examinations and the sudden aggravation of the patient's neurological state did not allow us to carry out this confirmatory assessment. Thus, in front of the impossibility of confirming or turning out the possibility of the HIT for this patient, the therapeutic process was challenging.

The choice to stop the treatment in this case was essentially based on the association of two elements: the aggravation of the neurological state, which could be related to a thrombosis extension, secondary to the HIT, and the reduction of platelets by more than 50% compared to previous figures. For patients with a moderate or high probability at the 4T score, it is recommended to break off any heparin exposure, even if the patient does not have a clinically obvious thrombosis. Because there is a significant risk of developing a thrombotic accident in the next 30 days. Hence, the patient should be put on an alternative anticoagulant [3].

## Conclusion

Anticoagulation is essential in the treatment of cerebral venous thrombosis. However, in rare cases, the occurrence of post-heparin therapy thrombocytopenia, leading to the suspicion of HIT, may be object of a therapeutic problem with respect to the discontinuation or continuation of the treatment, especially for the young child and the newborn baby. The diagnosis of the HIT should not be neglected, but it is also crucial not to wrongly conclude that it is present, especially in the absence of confirmatory elements, because the systematic discontinuation of heparin in the presence of thrombocytopenia causes therapeutic problems and can expose the patient to complications. The potential seriousness, the diagnostic and therapeutic difficulties of this pathology make the setting up of adapted recommendations for the child necessary.
